# Recurrent Contralateral Parotitis With Initial Neonatal Onset: A Rare Case Report

**DOI:** 10.7759/cureus.113776

**Published:** 2026-08-01

**Authors:** Noora Almuaili, Lateefa Almutawea, Emad Shatla, Salman M AlKhalifa

**Affiliations:** 1 Pediatrics, King Hamad University Hospital, Busaiteen, BHR; 2 Pediatrics and Neonatology, King Hamad University Hospital, Muharraq, BHR; 3 Pediatrics, King Hamad University Hospital, Manama, BHR; 4 Pulmonology, King Hamad University Hospital, Busaiteen, BHR

**Keywords:** bilateral parotitis, doppler imaging, neonatal parotitis, neonate, parotid gland infection, recurrent parotitis, salivary gland infection, staphylococcus aureus, suppurative parotitis, ultrasound

## Abstract

Neonatal parotitis is an uncommon bacterial infection of the salivary glands that may exhibit modest clinical manifestations, complicating early identification. This report details a case of an 11-day-old male neonate exhibiting left-sided facial edema and erythema, subsequently diagnosed with early parotitis.

The patient was an 11-day-old male neonate presenting with left-sided preauricular edema and agitation. A clinical examination revealed a solid, mildly uncomfortable swelling, with no purulence, emanating from Stensen's duct. Laboratory tests indicated elevated inflammatory markers, and blood cultures revealed the presence of *Staphylococcus aureus (S. Aureus)*. Ultrasound revealed that the left parotid gland was enlarged, exhibited a mixed echotexture, and displayed increased vascularity, indicative of early parotitis. The patient was administered intravenous antibiotics, resulting in a rapid improvement in their condition and total resolution on subsequent imaging. Two months later, he returned with right-sided facial swelling. Ultrasound findings were suggestive of right parotitis; however, inflammatory markers were normal, and cultures were negative. The patient exhibited a favorable response to supportive care, without any problems.

This example illustrates the utility of ultrasonography in detecting early or clinically mild instances of newborn parotitis. It also demonstrates an uncommon instance of recurring illness affecting the opposite side. Medical practitioners should be vigilant, especially in the absence of signs such as purulent discharges. Identifying difficulties promptly is crucial for achieving optimal results and preventing their recurrence.

## Introduction

Neonatal parotitis is a rare condition characterized by swelling, discomfort in the parotid region, irritability, and, less commonly, fever. It should be considered in the differential diagnosis of facial swelling, as the incidence of acute neonatal suppurative parotitis has been reported in limited epidemiological studies at roughly 3.8 per 10,000 neonatal admissions. Greater prevalence has been observed in preterm infants [1,2].

The bilateral occurrence of newborn parotitis is exceedingly uncommon. While the disease commonly affects neonates up to one month of age, the first two weeks of life are when it is most frequently noticed. The onset of symptoms is observed to be rapid and manifests within one to two days, with a documented predominance in male neonates [3,4].

The risk factors for infant parotitis comprise preterm birth, dehydration, congenital malformations of the parotid gland, oral trauma, and immunodeficiency. The predominant etiological agent of acute newborn suppurative parotitis is *Staphylococcus aureus (S. Aureus)*, constituting 55.2%, followed by Gram-positive and Gram-negative pathogens at 22.4% each, and anaerobic pathogens at 8.6%. *Escherichia coli* *(E. coli) *is an infrequent etiological agent [5,6].

The diagnosis of neonatal parotitis is primarily clinical; however, ultrasonography of the parotid gland aids in diagnosis and the exclusion of abscess formation. The majority of neonatal suppurative parotitis patients are effectively managed with antibiotics; nevertheless, instances of complications, including abscess formation or chronic infection, necessitate surgical intervention [7,8].

Potential complications of neonatal suppurative parotitis include osteomyelitis of the mandible or temporomandibular joint, jugular vein thrombophlebitis, respiratory obstruction, and sepsis, which must be considered for neonates experiencing a decline in condition. Facial palsy has been documented as a complication; nevertheless, the prognosis is typically favorable in cases of neonatal suppurative parotitis, given the administration of proper parenteral antibiotic therapy [9,10].

Acute neonatal parotitis is uncommon; however, it is easily diagnosable, necessitating immediate and suitable antibiotic combination therapy for the patient's swift recovery and to prevent sequelae. This report presents a case of an 11-day-old male neonate exhibiting left-sided facial edema and erythema, diagnosed with early parotitis.

## Case presentation

A male infant, 11 days old, presented to the pediatric emergency room with a 1-day history of left-sided facial edema and erythema localized to the pre-auricular region, extending downward toward the neck. He was born late-preterm at 36 weeks of gestation via cesarean section due to breech presentation with a birth weight of 2396 grams. Apgar scores were 9 and 10 at 1 and 5 minutes, respectively. The mother was a 28-year-old, known to have hypothyroidism managed on levothyroxine, with an unremarkable antenatal history and no prior antibiotic use. The patient did not require admission to the neonatal intensive care unit (NICU) and was discharged with the mother after delivery. Prior to presentation, the infant became increasingly distressed and cried excessively; however, there was no documentation of fever at home.

Upon admission, the patient's hemodynamics were stable. He developed a mild fever while under observation in the hospital. He received adequate nutrition every two hours from a combination of breast milk and formula, and there were no indications of dehydration.

The physical examination revealed a 1 × 1 cm mass in the left pre-auricular region extending to the upper neck. The edema varied from firm to soft, exhibited minor tenderness, and possessed a smooth, non-fluctuant surface. There was mild erythema in the skin above. No purulent discharge was observed from the Stensen duct during milking. The remainder of the examination of the ear, nose, and throat (ENT) region was unremarkable. The infant exhibited some jaundice in the chest region; nevertheless, the remainder of the systemic examination was unremarkable.

A comprehensive septic assessment was performed. Laboratory tests revealed an elevated C-reactive protein level of 33.5 mg/L, accompanied by leukocytosis at 27.88 × 10⁹/L and neutrophil predominance of 69.2%. The platelet count was elevated at 511 × 10⁹/L (Table 1). Blood culture grew *S. aureus,* demonstrating susceptibility to anti-staphylococcal beta-lactams, fluoroquinolones, clindamycin, trimethoprim-sulfamethoxazole (TMP-SMX), tetracycline, vancomycin, daptomycin, and linezolid, with resistance to penicillin. Concurrently, a urine culture grew *Klebsiella aerogenes (K. aerogenes) *at 10⁴ CFU/mL, which was susceptible to third- and fourth-generation cephalosporins, aminoglycosides, piperacillin-tazobactam, and carbapenems and resistant to ampicillin/amoxicillin-clavulanate, with intermediate fluoroquinolone susceptibility. The analysis of cerebrospinal fluid (CSF) indicated somewhat elevated protein levels, although it remained unremarkable, displaying negative culture and viral panel outcomes. No evidence of *Methicillin-resistant S. aureus *(MRSA​​​​​) was present.

**Table 1:** Episode 1: laboratory investigations findings

Test	Result	Normal reference values
C-reactive protein	33.5 mg/L	0 – 10 mg/L
White blood cells	27.88 × 10⁹/L	6 – 21 × 10⁹/L
Neutrophils percentage	69.2%	28 – 50 %
Neutrophil Absolute	19.29 × 10⁹/L	1.1 - 6.6
Hemoglobin	12.9 g/dL	10 – 14 g/dL
Platelets count	511 ×10⁹/L	150 – 450 ×10⁹/L
First blood culture	POSITIVE (Staphylococcus aureus)	-
Repeated blood culture	NEGATIVE	-
First urine culture	POSITIVE (Klebsiella Aerogenes)	-
Repeated urine culture	NO GROWTH	-

**Figure 1:**
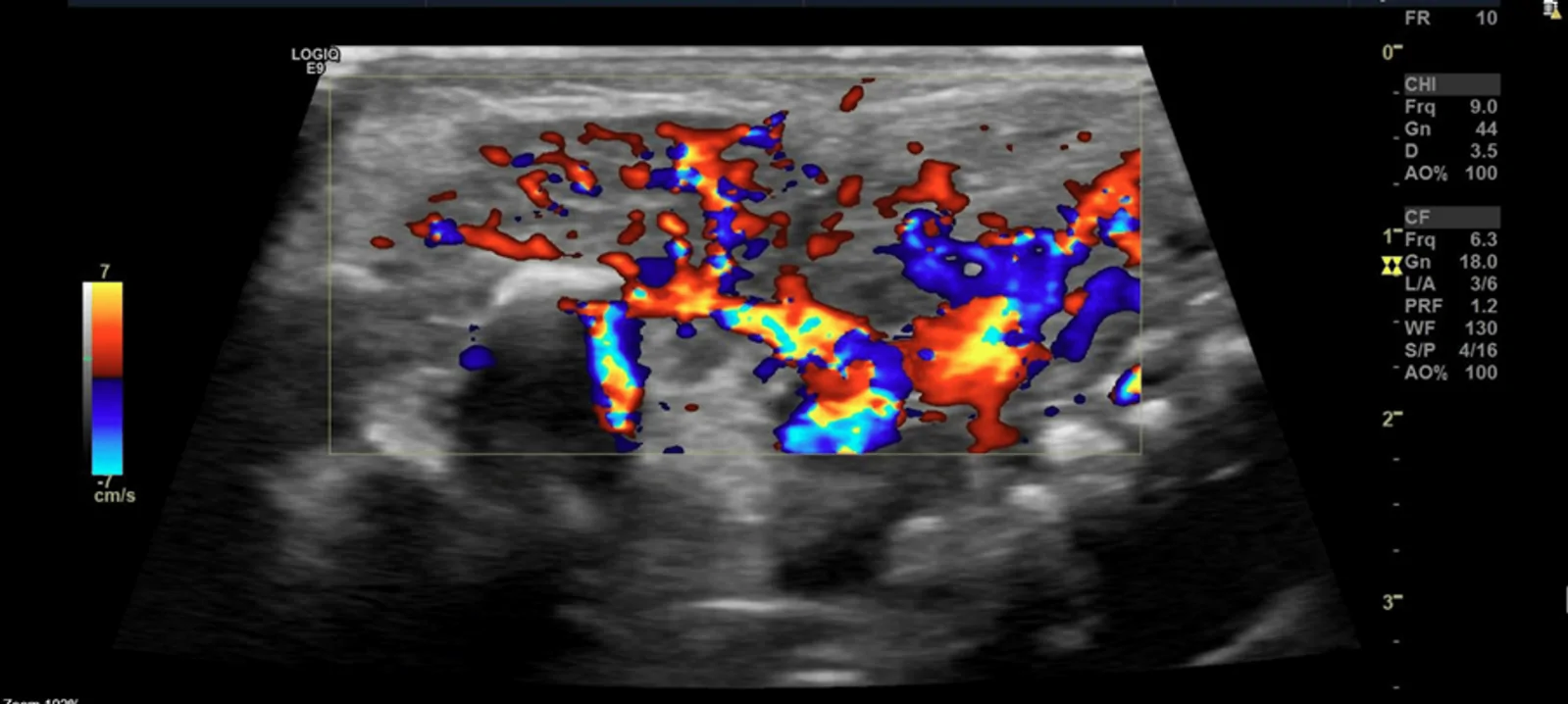
Ultrasound of the left parotid gland showing heterogeneous echotexture and increased vascularity on Doppler imaging, suggestive of early parotitis

An ultrasonography of the neck revealed that the left parotid gland was marginally enlarged and exhibited heterogeneous echotexture. The epidermis above it was merely a little thicker. Doppler imaging demonstrated increased vascularity, suggestive of early parotitis (Figure 1).

The patient received empirical intravenous antibiotics (ampicillin and cefotaxime) immediately. Subsequently, in consideration of culture and susceptibility profiles, the drugs were altered to vancomycin and amikacin to target both *S. aureus* bacteremia and the concurrent *K. aerogenes* urinary tract co-infection. A repeat ENT examination revealed normal oral mucosa and a negative milking test, with no discharge of pus.

Clinical improvement was observed within 48 hours, marked by the resolution of fever and reduction of edema. The patient completed a week-long course of intravenous antibiotics. The blood and urine cultures yielded negative results once more.

To objectively confirm complete resolution and to rule out any occult abscess prior to discharge, a follow-up ultrasound was performed on day 15 after the first presentation. The scan demonstrated normalization of the echotexture and vascularity of the left parotid gland, with minimal reactive intraparotid and preauricular lymph nodes and no signs of abscess or fluid collection.

Two months later, the patient was readmitted due to edema on the right side of the face, impacting the angle of the jaw. This was preceded by four days of subjective fever and upper respiratory symptoms. A firm, painful swelling of size 3 × 2 cm was present on the right jaw, with normal skin surrounding it. A discernible lymph node measuring 2 × 2 cm was seen in the right upper cervical region. No purulent discharge was observed from the Stensen duct. During this hospitalization, laboratory tests indicated normal levels of inflammatory markers (CRP 1.9 mg/L, ESR 3 mm/hr). The hemoglobin concentration was diminished (7.9 g/dL), but the platelet count was elevated (662 ×10⁹/L) (Table 2). No evidence of viruses or bacteria was detected in the blood cultures. Serological analysis for mumps (IgM) and immunoglobulin levels (IgG, IgM, and IgA) were both within normal limits (Table 2).

**Table 2:** Episode 2: laboratory investigations findings MRSA: Methicillin-resistant Staphylococcus aureus

Test	Result	Normal reference values
C-reactive protein	1.9 mg/L	0 – 10 mg/L
White blood cells	14.75 × 10⁹/L	6 – 21 × 10⁹/L
Neutrophils percentage	31.4 %	28 – 50 %
Neutrophil Absolute	4.63 × 10⁹/L	1.1 - 6.6
Hemoglobin	7.9 g/dL	10 – 14 g/dL
Platelets count	662 ×10⁹/L	150 – 450 ×10⁹/L
ESR (Erythrocyte Sedimentation Rate)	3 mm/hr	0 – 20 mm/hr
Blood culture	NEGATIVE	-
MRSA PCR nasal screening test	NEGATIVE	-
Immunoglobulin A (IgA)	< 33 mg/dl	87 - 474 mg/dl
Immunoglobulin G (IgG)	260.6 mg/dl	681 – 1648 mg/dl
Immunoglobulin M (IgM)	37.4 mg/dl	48 – 312 mg/dl
Anti-Mumps (IgM)	NEGATIVE	-

**Figure 2:**
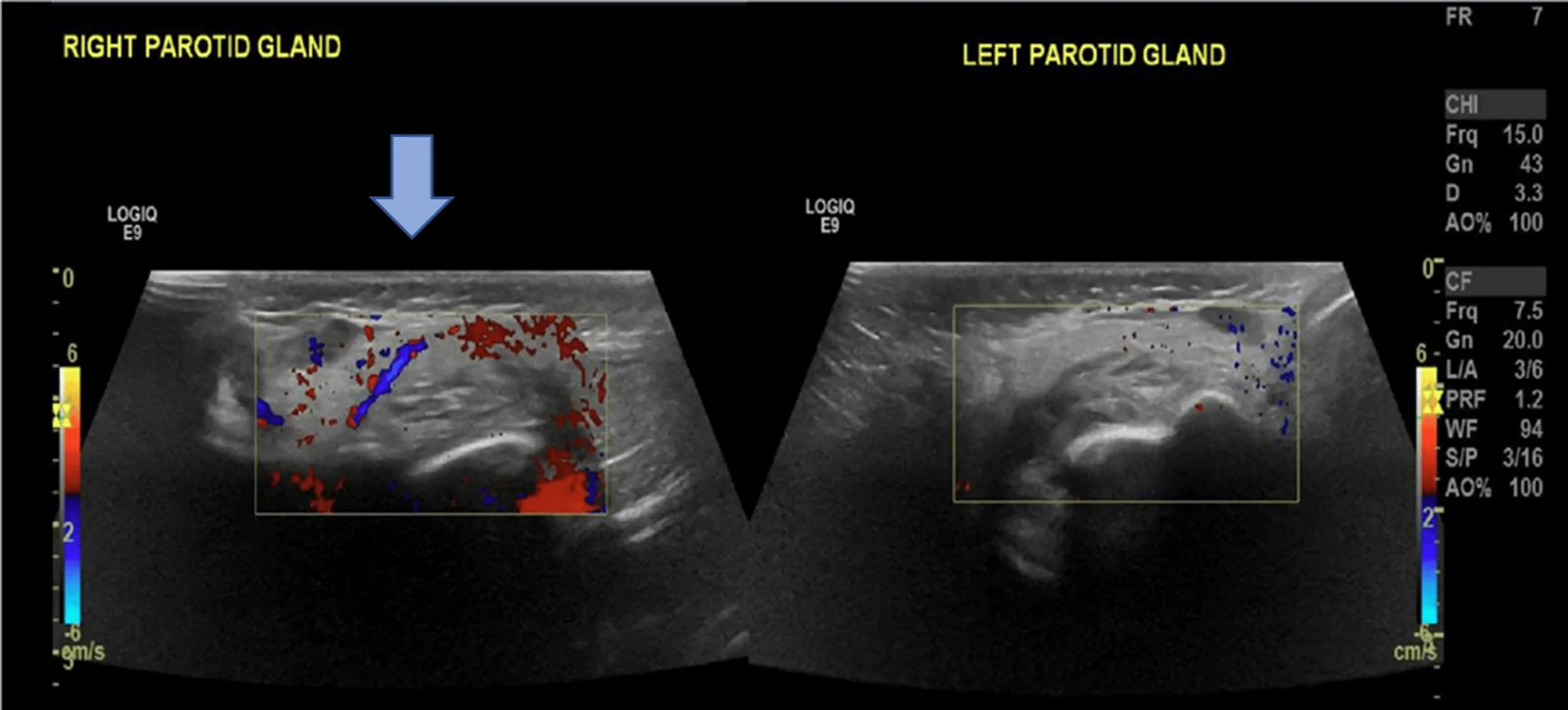
Ultrasound of the right parotid gland demonstrating increased vascularity and heterogeneous echotexture, consistent with recurrent parotitis

The ultrasonography revealed that the right parotid gland was enlarged and exhibited altered echotexture. It exhibited an increased number of blood vessels and reactive lymphadenopathy in the surrounding area, although there was no indication of fluid accumulation (Figure 2).

The patient was treated conservatively with intravenous fluids and antipyretic medicines. Following the negative culture findings, the antibiotics were discontinued. Within two days, the patient's condition significantly improved, and they were discharged with follow-up care.

The clinical, biochemical, and radiographic results were consistent with recurrent neonatal parotitis affecting each parotid gland at different intervals.

## Discussion

Neonatal parotitis is a rare but serious infection of the salivary glands caused by pathogens, including *S. aureus *[7]. However, facial edema in neonates may result from infectious, inflammatory, traumatic, vascular, congenital, or systemic conditions, while parotid swelling should prompt consideration of suppurative parotitis as well as non-infectious, obstructive, congenital, and neoplastic causes. The differential diagnosis of neonatal facial edema is broad and includes cellulitis, lymphadenitis, trauma or birth-related soft tissue injury, vascular or lymphatic malformations, congenital anomalies, allergic reactions, and systemic causes such as renal, cardiac, or hepatic disease associated with generalized edema. When facial swelling is localized to the parotid region, the differential diagnosis includes acute suppurative parotitis, viral parotitis (although uncommon in neonates), intraparotid abscess, dental abscess, ductal obstruction or sialolithiasis, congenital ductal anomalies, lymphadenopathy, hemangioma or lymphatic malformation, and, rarely, benign or malignant salivary gland tumors. Careful clinical examination, laboratory evaluation, and ultrasonography are essential to distinguish these conditions and establish the correct diagnosis.

Neonatal parotitis is considered a rare infection; however, it is a significant differential diagnosis in infants exhibiting facial edema or swelling anterior to the ears. Male neonates exhibit heightened vulnerability to neonatal parotitis, an inflammation of the parotid glands, attributable to dehydration, inadequate nutrition, immaturity, and the transmission of infection via Stensen's duct, the conduit responsible for transporting saliva from the parotid gland to the oral cavity. It is crucial to note that this bacterium can occur in healthy babies. Consequently, early detection is crucial to prevent complications such as abscess formation and septicemia [11,12].

The pathophysiology of neonatal parotitis is believed to entail retrograde bacterial contamination of the parotid gland via Stensen’s duct, worsened by reduced salivary flow or ductal stasis. Hematogenous dissemination has been proposed, particularly in cases associated with bacteremia. The isolation of *S. aureus *from blood culture indicates a possible hematogenous component in addition to a local ductal infection. This resembles other documented instances, such as the Lebanese case by Farah et al., in which MRSA was identified in ductal secretions and maternal origins. This research demonstrates both upward and exterior transmission routes [12].

Neonatal parotitis typically presents with unilateral facial edema, erythema, discomfort, and occasionally, fever. Nonetheless, it is widely acknowledged that the presentation may differ. The initial presentation was modest, with swelling more apparent upon palpation than visual inspection, highlighting the importance of thorough clinical assessment. Unlike some published cases, including those by Özdil et al. and El Omri et al., which exhibited significant fever and systemic toxicity, our patient initially displayed only minor systemic symptoms. This milder manifestation may result in a postponed diagnosis if clinicians rely solely on observable clinical indicators [13,14].

A notable clinical feature, as reported in the literature, is the discharge of pus from the duct upon massage, especially from Stensen's duct. This trait has been extensively recorded in the literature, particularly in the research by Farah et al. and Alfonso et al., where the expulsion of pus from the duct has been a critical element of the condition. Although the infection was verified, pus was not discharged from the duct. The lack of pus does not exclude the disease, particularly in the first phases or in milder instances [12,15].

Imaging, particularly by ultrasound, is an advantageous method to confirm the accuracy of the diagnosis and identify any potential issues. Ultrasound data typically indicate glandular enlargement, altered echotexture, and increased blood flow, particularly when employing Doppler technology. The ultrasound findings confirmed the diagnosis of early parotitis, marked by heightened vascularity, with no abscesses present. Özdil et al. and Alfonso et al. have previously recorded similar cases, noting hypervascularity and hypoechoic changes in the parotid glands. This case reflects the prior findings, demonstrating no disparity. Ultrasound was essential in detecting the problem and monitoring the status, especially in evaluating the reduction of inflammation. This study demonstrates the utility of this instrument [15,16].

The management of infant parotitis necessitates the immediate introduction of intravenous antibiotics effective against *S. aureus*, particularly MRSA. In this instance, empirical therapy was initiated promptly and then modified according to culture sensitivity results. This facilitated the patient's rapid recovery. The literature has recorded a consistent approach to managing parotitis, utilizing vancomycin, amikacin, and cephalosporins. Surgical procedures are generally reserved for patients with abscess formation, as illustrated in the case by Alfonso et al. [15], which underscores the efficacy of surgical treatment in these situations.

Because our patient developed recurrent contralateral parotitis, evaluation for underlying predisposing conditions was warranted. Potential causes include repeated bacterial infections, obstructive or congenital salivary duct abnormalities, juvenile recurrent parotitis, primary immunodeficiency, and autoimmune disease. The workup should include microbial cultures when feasible, baseline laboratory testing and immunological evaluation, and ultrasonography as the first-line imaging study. Computed tomography (CT), magnetic resonance imaging (MRI), or sialography may be considered only in selected cases when anatomical detail is needed [2,7,12].

An interesting element of this case is the onset of recurring parotitis in the opposite gland following a two-month period. Reports of bilateral parotitis have suggested its occurrence either concurrently or during the same hospitalization, as recorded by Farah et al. and Miranda et al. In this case, however, there was a sequential occurrence with a phase of clinical remission distinguishing the two episodes. Thus, it exemplifies a rare case of recurrent newborn parotitis. This raises inquiries regarding host susceptibility, despite normal immunological tests for this patient and others, indicating potential unrecognized underlying causes such as genetic predispositions or environmental triggers that remain inadequately explored [12,16].

A notable characteristic of this case is the discrepancy in the inflammatory response between the two occurrences. The initial episode was associated with marked elevation of inflammatory markers and positive microbial cultures. In contrast, the second episode was characterized by normal inflammatory markers, negative cultures, and the absence of purulent discharge. Although the clinical presentation and radiological findings were suggestive of parotid involvement, these findings limit the certainty of recurrent bacterial infection. The second episode may have represented a non-infectious inflammatory condition or a milder infectious process. To our knowledge, this trait was not noted in other cases [16].

This case has significant clinical implications, including the potential for mild presentation, the utility of ultrasound in early detection, and recurrence with contralateral involvement. This case contributes to existing knowledge by demonstrating a rare instance of successive bilateral involvement with varying levels of clinical presentation, despite the overall commonality of the presentation. This highlights the necessity of vigilant observation of infants afflicted with this condition [12-16].

## Conclusions

Neonatal parotitis is an uncommon ailment, and it is crucial to recognize it in neonates exhibiting signs, such as facial and periauricular edema, regardless of their severity. This example demonstrates that ultrasound imaging is essential for diagnosis, particularly when symptoms are ambiguous such as edema and purulent discharge. The patient may experience immediate improvement following diagnosis and administration of antibiotics. Such measures can prevent significant issues from arising. This clinical case highlights that recurrent and contralateral parotitis, although rare, may occur post-treatment. Evaluation for underlying etiologies, such as immunological and salivary duct abnormalities, should be considered in such cases.
